# Assessing the level of evidence on transfer and transition in young people with chronic conditions: protocol of a scoping review

**DOI:** 10.1186/s13643-016-0344-z

**Published:** 2016-09-29

**Authors:** Mariela Acuña Mora, Philip Moons, Carina Sparud-Lundin, Ewa-Lena Bratt, Eva Goossens

**Affiliations:** 1 Institute of Health and Care Sciences, Sahlgrenska Academy, University of Gothenburg, Box 457, Gothenburg, SE-405 30 Sweden; 2 Department of Public Health and Primary Care, Academic Center for Nursing and Midwifery, KU Leuven, Kapucijnenvoer 35, box 7001, 3000 Leuven, Belgium; 3 Department of Pediatric Cardiology, The Queen Silvia Children’s Hospital, Gothenburg, Sweden; 4 Research Foundation Flanders (FWO), Brussels, Belgium

**Keywords:** Transition, Transfer, Transition to adulthood, Chronic condition, Pediatric, Care provision, Scoping review

## Abstract

**Background:**

Life-long specialized care is of the utmost importance to safeguard longevity as well as the quality of life in children diagnosed with a chronic condition (CC). Provision of life-long care, however, infers transfers to different settings in line with person’s development status. Young people with CC (10–25 years) will transfer care from a pediatric towards an adult-oriented care setting. As a transfer of care is associated with a change of care context, healthcare team, responsibilities, expectations, and roles, patients need to be prepared for this alteration. One type of preparatory intervention is the provision of transitional care. Transition prepares adolescents for the responsibilities associated with adult care and age through support, education, skills demonstration, and guidance. The past decades, increasing attention has been paid towards the concept of transfer and transition, both in clinical practice and research. Numerous consensus papers have been established, emphasizing the need for the establishment of a transition program for young patients with CC. To date, it remains, however, unclear what the overall level of evidence is on transfer and transition in this population. This scoping review aims to analyze and determine the level of evidence of published literature on transfer and transition of young people diagnosed with CC.

**Method:**

MEDLINE, CINAHL, Scopus, and Web of Science databases will be searched for relevant publications. Any publication in English, Spanish, German, or French, related to transfer and/or transition in young people with CC will be included. A three-staged approach will identify relevant papers, comprising systematic database searches, application of snowball method, and citation searching. Study selection will be performed through screening of titles/abstracts followed by a full-text assessment using a standardized selection form. Data extraction will be performed by two reviewers independently using a pilot-tested, standardized form. Descriptive statistics and content analysis will be applied to present the results. Bibliometric visualization techniques will be performed with VOS viewer®.

**Discussion:**

Our review will map the overall level of evidence of published literature on transfer and transition in young people with CC. It will provide guidance for future research initiatives, clinical practice, and policy makers.

**Electronic supplementary material:**

The online version of this article (doi:10.1186/s13643-016-0344-z) contains supplementary material, which is available to authorized users.

## Background

About four decades ago, children with a chronic condition (CC) had a short life expectancy. For example, children with cystic fibrosis had a mean survival age of 7 years [1], and children with complex cardiac malformations had an average life expectancy of 2 years [2]. Improvements in diagnostic techniques and treatment of CCs in children have led to an increased survival of this population [3, 4]. Nowadays, more than 90 % of these children are expected to reach adulthood [5–7]. This resulted in an increased prevalence of chronic conditions. Indeed, nowadays up to 40 % of children live with one or more congenital and/or chronic condition, representing the wide spectrum of mild to severe conditions [8].

Advances in patients’ prospects bring new challenges for the healthcare system. Since the majority of young people with CCs are in need of life-long specialized care, transfers over different care settings along the life spectrum of patients are mandatory.

Such transfers aim to provide patients with continuous and age-appropriate care, which addresses the actual patient needs. Young patients affected with CCs are recommended to transfer from pediatric to adult care. This transfer of care is defined as an “event or series of events through which adolescents and young adults with chronic physical and medical conditions move their care from pediatric to an adult care environment” ([9], p. 292). This process ought to be coordinated between healthcare providers and patients, guaranteeing continuity of specialized care in accordance with individuals’ age and developmental level.

Transition is a concept that is related to this transfer of care. Transition is defined as the “process by which adolescents and young adults with chronic childhood illnesses are prepared to take charge of their lives and their health in adulthood” ([9], p. 291). Adolescents affected by CCs are considered to go through both a developmental and health/illness transition simultaneously. The developmental transition refers to the gradual developmental change from adolescence towards adulthood [10], whereas the health/illness transition represents the actual change of a patient from a pediatric to an adult-focused setting, denoting the transfer of care [10]. In order to guarantee a seamless transfer between care settings when patients with CCs grow older, several strategies and interventions, tailored to the needs of this population, are recommended by task forces, consensus statements, and policy documents [11].

The number of studies performed in the area of transfer and transition of care has increased over the past decade. However, it is unclear which specific aspects of transfer and/or transition have been the focus of such research initiatives. A comprehensive overview and mapping of the existing literature on transfer and transition will provide details on the content and magnitude of the evidence base, as well as help identifying gaps in our understanding. A variety of studies have been conducted previously, including studies that investigated adolescents’ needs and experiences during transfer [12–14], the role of healthcare professionals [15, 16], and the impact of transition programs on patient-reported outcomes [17–19]. Some of the available evidence has been synthesized into literature reviews which focused on specific aspects of transfer, such as patients’ perspectives and experiences on transfer [20, 21], determinants of care gaps [22], and effectiveness of practices that improve continuity of care [23–26].

Although these latter reviews often stated to review evidence regarding transitional care of young people with CC, they were actually summarizing the evidence base on a diversity of aspects related to transfer of care exclusively [23, 26]. While transfer and transition are concepts related to one another, they cannot be used interchangeably. Hence, a comprehensive assessment of the state of research on either transfer, either transition or both concepts is currently lacking. Through this review, it will be possible to distinguish the evidence base related to transfer from the evidence related to transition of care. Moreover, the scope of this review will help us to map the existing evidence base on transfer and/or transition in relation to all types of chronic conditions through a descriptive analysis of study characteristics.

### Objectives

As a primary objective, this scoping review aims to determine the type, amount, and evidence level of published literature on transfer and/or transition of young people diagnosed with a chronic condition. A secondary objective is to describe the characteristics of published studies on transfer and transition using descriptive statistics, content analysis, and visualization techniques.

## Methods/design

### Protocol development

A scoping review protocol was developed based on the instructions provided by the Preferred Reporting Items for Systematic Reviews and Meta-Analyses Protocols (PRISMA-P) statement [27], and a PRISMA-P protocol checklist was used while developing this review protocol (see Additional file 1). This review protocol was submitted to the PROSPERO register but could not be registered since our review will not report intervention effects but will assess the level of evidence.

### Eligibility criteria

Study selection will be based primarily on population and relevance to transfer and transition of care.

#### Types of study participants

The population of interest within this scoping review is children and young people (aged 10–25 years) who are afflicted by a chronic condition requiring life-long specialized care. The broad range of medical and psychosocial conditions with a congenital and/or chronic nature will be included in this scoping review, ranging from somatic to psychological and neurodevelopmental conditions. This group of conditions can be defined as “any medical condition that can be reasonably expected to last at least 12 months (unless death intervenes) and to involve either several different organ systems or one organ system severely enough to require specialty pediatric care and probably some period of hospitalization in a tertiary care center” ([28], p. 206).

#### Type of studies

Since this review primarily aims to determine the amount, type, and evidence level of studies on transfer and transition, all the published studies using any type of study design will be included. Gray literature (e.g., conference proceedings, theses, working papers, and research reports) will, however, not be included. The design of every respective study, as well as the type of publication, will be determined using a standardized form (see Additional file 2). Quantitative, qualitative, and mixed method studies will be included. Publications written in Spanish, English, German, or French with any content related to any aspect of transfer and/or transition of young people (aged 10–25 years) with a chronic condition will be included. No restrictions in terms of publication dates will be applied.

Studies are eligible for inclusion if they address aspects of transfer and/or transition of care as defined by Meadows (definitions provided in the “Background” section) [9]. Information in existing guidelines regarding transfer and transition will also help define these concepts accordingly. Furthermore, the research team involved in the review has extensive experience in the area of transfer and transition of care which facilitates the assessment of issues that arise in relation to this process. Studies are eligible if they address some aspect(s) of transfer and/or transition, studies can for example be related to, although not be limited to primary studies and systematic reviews examining the following:

- Young persons, parents, and healthcare providers’ views and experiences regarding transfer and/or transition
- Interventions designed to facilitate transfer and/or transition
- Determinants of transfer and/or transition
- Development of transfer and/or transition related measurements

#### Defining characteristics of transition and transfer

Included studies will be categorized based on the focus of their research question(s), which are either related to transition exclusively, transfer exclusively, or both concepts. This categorization will be based on the following set of criteria:

- Transition refers to the process followed by young persons with chronic conditions in order to take charge of their health and lives in adulthood [9]. This process entails interventions, such as providing structured and repetitive patient education, counseling services of patients regarding lifestyle issues and health behaviors, and care and skills demonstration provided by transition coordinators (e.g., self-management, participation in care, self-efficacy, communication, decision making) [7–9, 11].
- Transfer is, in general, a single occasion event through which the young patient is transferred from pediatric to adult care services. Examples of transfer-related interventions are the following: writing a transfer letter, developing a transfer plan, writing a health summary and coordinated transfer of care [9].

#### Search strategy

The databases of MEDLINE (PubMed), CINAHL, Scopus, and Web of Science - Core Collection™ will be searched for relevant publications using a predefined search string. An example of the MEDLINE search string can be found in Additional file 3.

A three-staged approach will be used to identify relevant publications. In a first step, an electronic search will be performed in each of the four electronic databases using a standardized search string which is converted to the specifications of every respective database. A second step will subsequently entail the application of the snowball method, a screening technique applied on the reference lists of relevant publications retrieved at the first step. Finally, a third step includes citation searching of key publications in Web of Science - Core Collection™ and Scopus.

#### Study selection

Subsequently, a two-staged approach will be used for the selection of relevant publications. The first stage entails the initial screening of all retrieved references based on their title and/or abstract against the abovementioned in- and exclusion criteria to identify potentially relevant publications. In a second stage, the full-text version of selected references from stage 1 will be assessed for eligibility using a standardized selection form (see Additional file 4). This selection form will be used to collect data on the reason(s) for exclusion of papers that were selected based on title and/or abstract. Both stages will be performed by two reviewers independently (EG and MAM). Any disagreement related to the eligibility of the publications will be resolved during a face-to-face discussion and if, agreement is not reached, a third member of the review team will intervene. The results of this staged selection process will be documented in a standardized PRISMA flowchart [29].

#### Data extraction and management

The data extraction process will be performed by two reviewers independently (EG and MAM) using a standardized data extraction form (see Additional file 5). Any disagreement related to the data extraction will be resolved during a face-to-face discussion and if agreement is not reach, a third member of the review team will intervene. The data extraction form was developed by two reviewers (EG and MAM) and was pilot tested by five review team members independently on a selection of 20 different publications. In order to support the reviewer team during the data extraction process in determining the specific research design of a publication, a flowchart of study designs (see Additional file 6) and a glossary with definitions of study designs from either quantitative, qualitative, or mixed methods design (see Additional file 2) were developed, pilot tested, and implemented during the review process.

The assessment of the level of evidence of included studies involves categorizing each study design according to the “New Joanna Briggs Institute (JBI) Levels of Evidence” developed by the JBI Levels of Evidence and Grades of Recommendation Working Party (Oct 2013) [30]. No specific findings or results from individual studies will be extracted, reported, nor synthesized for the purpose of this review. Full details on the data that will be extracted from the included studies can be found in the data extraction form (Additional file 5).

EndNote X7® software will be used to manage retrieved references from the respective electronic database. This software will enable us to: (i) identify and remove duplicates; (ii) perform, manage, and document the screening process of titles and abstracts of retrieved references; (iii) to categorize publications that will be in- or excluded from the bibliometric review, and (iv) to manage full text versions of the publications. Additional notes or remarks can be added and shared between all review team members. For each relevant publication, a completed data extraction form will be added in attachment in this Endnote X7® library.

#### Data synthesis and analysis

Descriptive statistics will be applied to fully describe the characteristics of the published literature. Statistical analysis will be performed using SPSS Version 24.0. An identified literature will be described in terms of number of publications per year; language in which papers are published; region(s) and countr(y)(ies) in which the study was performed (depending on type of publication); research groups performing studies; characteristics of study population with sample size; and study design and corresponding level of evidence. These descriptive results will be presented in tables and graphs. Detailed information on the types of outcomes, findings and risk of bias of the included studies will, however, not be reported.

Bibliometric visualization techniques will be used to construct and visualize bibliometric networks, clustering of papers, and regions of publications. The software tool of VOSviewer® will be used for these visualizations. Finally, a content analysis will be performed on the respective research questions addressed in the included publications. This content analysis aims to define which aspect(s) of transfer and/or transition the study is addressing, identifying common themes, clustering of objectives and gaps in our understanding of transfer and transition [31]. The results of this content analysis will be structured based on emerging themes.

## Discussion

This scoping review entails an analysis of the published literature on transfer and transition in a population of young people with a chronic condition. Due to an increasing interest for the concepts of transfer and transition, predominantly in pediatric clinical practice and research initiatives, the number of publications on these issues was observed to be increasing remarkably over the past years. This review will analyze the characteristics of these publications and determine if the increasing number of papers found in literature is associated with an increasing level of evidence on transfer and transition. Based on the insights gained in this review, focused suggestions for future research initiatives and study designs addressing the identified gaps in the existing body of literature can be given.

Although a sound methodology was developed for the performance of this scoping review, we anticipate several sources of bias. First, a language bias will be inevitable since only papers published in English, French, German, or Spanish will be included in this review. Furthermore, publication bias will be present as well since gray literature will be excluded from this review.

Since the aim of this review is to provide an overview of the existing body of the published literature, the search string was developed to maximize recall ensuring to capture all relevant publications. Unfortunately, high recall is often associated with a lower precision which might result in a large number of redundant references which increases the work load for the review team [32]. However, these irrelevant publications will be excluded at stage 1.

Finally, a significant amount of time and effort was put in the development of the search string, standardized selection and data collection forms. It is important to mention that our search strings were developed in collaboration with two expert librarians. Although the content and structure of these documents were thoroughly assessed by the review team during a pilot-testing phase, these documents were established before the start of the review process. During the data collection process, some refinement of study selection and data collection tools may be required with the aim of improving the quality, consistency and structure of data extraction.

## Additional files

Additional file 1: PRISMA—protocol checklist. Additional file 2: Glossary of study designs. Additional file 3: Example of MEDLINE search string. Additional file 4: Standardized selection form. Additional file 5: Standardized data extraction form. Additional file 6: Flowchart of study designs.

## Abbreviations

CC: Congenital and/or chronic condition
JBI: Joanna Briggs Institute
PRISMA-P: Preferred Reporting Items for Systematic Reviews and Meta-Analyses Protocols
