# Supplementary material for: Assessing the level of evidence on transfer and transition in young people with chronic conditions: protocol of a scoping review
Source: Syst Rev. 2016 Sep 29;5:166. doi: 10.1186/s13643-016-0344-z (PMC5043611; doi:10.1186/s13643-016-0344-z)
Supplement: Additional file 2: — Glossary of study designs. [file 13643_2016_344_MOESM2_ESM.docx]

**Additional file 2: Glossary of study designs**

# ****GLOSSARY Study designs*****

**QUANTITATIVE DESIGNS (level of evidence according to JBI)**

**LEVEL 1– Experimental designs**

**□ level 1.a: systematic review of RCTs = meta-analysis**

**→ *systematic review of RCT’s = systematic allocation, appraisal and synthesis of evidence from scientific studies, only including RCT’s***

**□ level 1.b: systematic review of RCTs and other study designs**

**→ *systematic review of RCT’s and other study designs = systematic allocation, appraisal and synthesis of evidence from scientific studies, including both RCT’s and other study designs***

**□ level 1.c: RCT**

**→ *RCT = the unit of experimentation (e.g., people or cluster of patients) is allocated to either an intervention group or a control group, using a random mechanism (e.g., coin toss, random number table, computer-generated random numbers) and the outcomes from each group are compared*.**

**□ level 1.d: Pseudo-RCT**

**→ *pseudo – RCT = the unit of experimentation (e.g., people or cluster of patients) is allocated to either an intervention group or a control group, using a pseudo-random method (e.g., alternate allocation, allocation by days of the week or odd-even study numbers) and the outcomes from each group are compared.***

**LEVEL 2 – Quasi-experimental designs**

**□ level 2.a: systematic review of quasi-experimental studies**

**→ *systematic review of quasi-experimental studies = systematic allocation, appraisal and synthesis of evidence from scientific studies, only including quasi-experimental studies***

**□ level 2.b: systematic review of quasi-experimental and other lower study design**

**→ *systematic review of quasi-experimental studies and other designs = systematic allocation, appraisal and synthesis of evidence from scientific studies, including both quasi-experimental studies and other research designs***

**□ level 2.c: quasi-experimental prospectively controlled study**

**→ *quasi-experimental study = the unit of experimentation (e.g., people or cluster of patients) is allocated to either an intervention group or a control group, using a non- random mechanism (e.g., e.g., patient or physician preference) and the outcomes from each group are compared.***

**□ level 2.d: Pre-test/Post-test or historic/retrospective control group study**

**→ *historic/retrospective control group study = outcomes for a prospectively collected group of patients exposed to the intervention are compared with either (1) the outcomes of patients treated at the same institution prior to the introduction of the intervention, or (2) the outcomes of a previously published series of patients undergoing the alternate or control intervention***

**LEVEL 3 – Observational-analytic designs**

**□ level 3.a: systematic review of comparable cohort studies**

**→ *systematic review of comparable cohort studies = systematic allocation, appraisal and synthesis of evidence from scientific studies, only including cohort studies***

**□ level 3.b: systematic review of comparable cohort and other lower study design**

**→ *systematic review of comparable cohort studies and other designs = systematic allocation, appraisal and synthesis of evidence from scientific studies, including both cohort studies and other research designs***

**□ level 3.c: cohort study with control group**

**→ *cohort study = outcomes for groups of patients who are to be exposed to an intervention, or the factor under study, are compared to outcomes for groups of patients not exposed.***

**Can be a prospective cohort study or a retrospective cohort study:**

**→ *prospective cohort study = where groups of patients (COHORTS) are observed at a point in time to be exposed or not exposed to an intervention and then are followed prospectively with further outcomes recorded as they happen.***

**→ *retrospective cohort study = where the groups of patients (COHORTS) are defined at a point of time in the past and information collected on subsequent outcomes.***

**□ level 3.d: case-controlled study**

**→ *case-controlled study = people with the outcome or disease (CASES) and an appropriate group of controls without the outcome or disease (CONTROLS) are selected and information obtained about their previous exposure/non-exposure to the intervention or factor under study.***

**□ level 3.e: observational study without control group**

**→ *observational study without control group = study without experimental features aiming to summarize associations between variables in order to generate (rather than to test) hypotheses. These studies are solely based on observing what happens or what has happened. Observational studies can be broadly described as being either Correlational or Descriptive*.**

**LEVEL 4 – Observational-descriptive designs**

**□ level 4.a: systematic review of descriptive studies**

**→ *systematic review of descriptive studies = systematic allocation, appraisal and synthesis of evidence from scientific studies, only including both descriptive studies***

**□ level 4.b: cross-sectional study**

**→ *cross-sectional study = a group of patients are assessed at a particular point in time and the data collected on outcomes relate to that same point in time*.**

**□ level 4.c: case series**

**→ *case series = a group or series of case reports involving patients who were given similar treatment. Reports of case series usually contain information about individual patients. This includes demographic information (e.g., age, sex, ethnic origin) and information on diagnosis, treatment, response to treatment, and follow-up after treatment.***

**□ level 4.d: case study**

**→ *case study = a description of a single case***

**LEVEL 5 – Expert opinion and bench research**

**□ level 5.a: systematic review of expert opinion; narrative review**

**→ *systematic review of expert opinion = systematic allocation, appraisal and synthesis of evidence from scientific studies, only including expert opinions***

**□ level 5.b: expert consensus**

**→ *expert consensus = evidence arising from the consensus of experts in the field***

**□ level 5.c: bench research/single expert opinion**

**→ *bench research = studies that have been conducted on non-human subjects in a laboratory setting.***

**QUALITATIVE DESIGNS**

**□ Phenomenology**

**→ *description of how individuals experience a phenomenon***

**□ Etnography**

**→ *description of the culture of a group of people***

**□ Case studies**

**→ *detailed account and analyses or one or more cases***

**□ Grounded theory**

**→ *development of inductive bottom up theory that is “grounded” directly in empirical data***

**** Based on The Joanna Briggs Institute Levels of Evidence and Grades of Recommendation Working Party. Supporting Document for the Joanna Briggs Institute Levels of Evidence and Grades of Recommendation. The Joanna Briggs Institute. 2014. (***[***http://joannabriggs.org/assets/ docs/approach/Levels-of-Evidence-SupportingDocuments-v2.pdf***](http://joannabriggs.org/assets/%20docs/approach/Levels-of-Evidence-SupportingDocuments-v2.pdf)***, accessed on March 3, 2016)***
