# Supplementary material for: Assessing the level of evidence on transfer and transition in young people with chronic conditions: protocol of a scoping review
Source: Syst Rev. 2016 Sep 29;5:166. doi: 10.1186/s13643-016-0344-z (PMC5043611; doi:10.1186/s13643-016-0344-z)
Supplement: Additional file 3: — Example of MEDLINE search string. [file 13643_2016_344_MOESM3_ESM.docx]

**Additional File 3: Example of MEDLINE search string**

child*[Title/Abstract]) OR adolescen*[Title/Abstract]) OR pediatric*[Title/Abstract]) OR paediatric*[Title/Abstract]) OR adult*[Title/Abstract]) AND (((((((((((((continuity of patient care [MeSH Terms]) OR "continuity of patient care") OR "transfer to adult care") OR "transfer of care") OR healthcare transition*) OR "continuity of care") OR transitional care[Title/Abstract]) OR "gaps in care") OR "transition care") OR transition to adulthood[Title/Abstract]) OR transition readiness[Title/Abstract]) OR transition program*) OR health care transition*[Title/Abstract] OR transition from pediatric[Title/Abstract]) OR transition from paediatric [Title/Abstract]) OR transition to adult[Title/Abstract]) OR transition from adolescence[Title/Abstract]
