# Supplementary material for: Assessing the level of evidence on transfer and transition in young people with chronic conditions: protocol of a scoping review
Source: Syst Rev. 2016 Sep 29;5:166. doi: 10.1186/s13643-016-0344-z (PMC5043611; doi:10.1186/s13643-016-0344-z)
Supplement: Additional file 4: — Standardized selection form. [file 13643_2016_344_MOESM4_ESM.docx]

**Additional file 4: Standardized Selection Form**

**STARDARDIZED SELECTION FORM**

*Systematic review: level of evidence on transfer and transition in young people with a chronic condition*

| **GENERAL INFORMATION** |
| --- |
| **Study ID:** |
| **Reviewer: □** Mariela □ Eva □ Other: |
| **Date**: / /2016 |
| **SCREENING OF FULL-TEXT ARTICLES** |
| **Reasons for exclusion:**  □ Full text is not available  □ Full text available in a language that is not English, Spanish, French, German and Dutch  -Indicate the language in which the article is written: ________________________  □ Study sample did not include patients with a chronic and/or congenital condition  □ Study sample did not include young people (age 10-25 years)  □ Subject of the paper is not related to transfer and/or transition (as defined within the context of the systematic review)  □ Qualifies as grey literature publication (i.e. newsletters, preprints, conference proceedings)  □ Studies based on data previously published in other studies included in the review  □ Other reasons for exclusion: ______________________________________________________  _______________________________________________________________________________ |
| **Additional comments:** |
