# Supplementary material for: Assessing the level of evidence on transfer and transition in young people with chronic conditions: protocol of a scoping review
Source: Syst Rev. 2016 Sep 29;5:166. doi: 10.1186/s13643-016-0344-z (PMC5043611; doi:10.1186/s13643-016-0344-z)
Supplement: Additional file 5: — Standardized data extraction form. [file 13643_2016_344_MOESM5_ESM.docx]

**Additional file 5: Standardized Data Extraction Form**

| **GENERAL INFORMATION** | | | | | | |
| --- | --- | --- | --- | --- | --- | --- |
| **Study ID:** | | | | | | |
| **Reviewer** | **□ Mariela** | **□ Eva** | | | **□ other:** | |
| **Date: ..../…./2016** | | | | | | |
| **First author:** | | | | **Last author:** | | |
| **Journal:** | | | | **Year of publication:** | | |
| **Study was performed in (continent and country):** | | | | | | |
| **□ Africa:**  **□ North- America:**  **□ South-America:**  **□ Antarctica:**  **□ Asia:** | | | **□ Oceania:**  **□ Europe:**  **□ International:**  **□ Not applicable** | | | |
| **STUDY POPULATION** | | | | | | |
| **Diagnosis and sample size *(multiple options are possible)*:** | | | | | | |
| **□ auto-immunology:**  **□ cardiology:**  **□ dermatology:**  **□ developmental condition(s):**  **□ endocrinology:**  **□ gastro-enterology:**  **□ hematology:**  **□ mental health issues:**  **□ nephrology:**  **□ neurology:**  **□ oncology:**  **□ organ transplantation:**  **□ pneumology:**  **□ rheumatology:**  **□ system disease:**  **□ Special Health Care Needs (SHCNs)**  **→*definition provided in paper + REFERENCE of definition*:**  **□ other condition(s):** | | | | | | **n=**  **n=**  **n=**  **n=**  **n=**  **n=**  **n=**  **n=**  **n=**  **n=**  **n=**  **n=**  **n=**  **n=**  **n=**  **n=**  **n=** |

| **METHODOLOGY** | |
| --- | --- |
| **Research aim:** | |
| **Study design:** | **□ QUALITATIVE DESIGN**  **□ grounded theory**  **□ phenomenology/ hermeneutics**  **□ case study**  **□ ethnography**  **□ other:** |
|  | **□ QUANTITATIVE DESIGN *(level of evidence according to JBI)***  **└ LEVEL 1– Experimental designs**  **□ level 1.a: systematic review of RCTs = meta-analysis**  **□ level 1.b: systematic review of RCTs and other study designs**  **□ level 1.c: RCT**  **□ level 1.d: Pseudo-RCT**  **└ LEVEL 2 – Quasi-experimental designs**  **□ level 2.a: systematic review of quasi-experimental studies**  **□ level 2.b: systematic review of quasi-experimental and other lower study design**  **□ level 2.c: quasi-experimental prospectively controlled study**  **□ level 2.d: Pre-test/Post-test or historic/retrospective control group study**  **└ LEVEL 3 – Observational-analytic designs**  **□ level 3.a: systematic review of comparable cohort studies**  **□ level 3.b: systematic review of comparable cohort and other lower study design**  **□ level 3.c: cohort study with control group**  **□ level 3.d: case-controlled study**  **□ level 3.e: observational study without control group**  **└ LEVEL 4 – Observational-descriptive designs**  **□ level 4.a: systematic review of descriptive studies**  **□ level 4.b: cross-sectional study**  **□ level 4.c: case series**  **□ level 4.d: case study**  **└ LEVEL 5 – Expert opinion and bench research**  **□ level 5.a: systematic review of expert opinion; narrative review**  **□ level 5.b: expert consensus/ guidelines/consensus statements/task forces**  **□ level 5.c: bench research/single expert opinion**  **□OTHER Type of publication/study:………………………………** |
|  | **□ MIXED METHODS DESIGN** |

| **DATA COLLECTION METHOD (*multiple options possible*):** | | | | | |
| --- | --- | --- | --- | --- | --- |
| **□ questionnaire(s)/survey/instrument(s)/scale(s)**  **□ individual face-to-face interviews**  **□ focus groups**  **□ chart review/ medical file review/ electronic hospital records**  **□ observations**  **□ field notes**  **□ registered data / existing database(s)**  **□ other:**  **□ not applicable due to the type of publication** | | | | | |
| **Focus of study/ research objective(s):**  **□ on transfer exclusively → RQ:**  **□ on transition exclusively → RQ:**  **□ on both transfer and transition → RQ:** | | | | | |
| **STUDY POPULATION *(multiple options are possible)*: □ *this section is not applicable for this paper*** | | | | | |
| **□ patient(s) n=** | **□ parent(s) n=** | | | **□healthcare professional(s) n=** | **□ other: n=** |
| **Age of participants:** | | | | | |
| **age range: [........y -.........y]**  **mean : .........y ± ...........y (SD)**  **median :………y ;Q_1_…y-Q_3_…y** | | | **□ not reported □ notes:**  **□ not reported □ notes:**  **□ not reported □ notes:** | | |
| **Ethnicity:** | | **□ White, Caucasian (……%; n= )**  **□ Black, African-American (……%; n= )** | | | |
|  |  | **□ Middle Eastern / Arabic (……%; n= )** | | | |
|  |  | **□ Asian (……%; n= )** | | | |
|  |  | **□ Hispanic / Latino (……%; n= )** | | | |
|  |  | **□ Other: ……………………… (……%; n= )**  **□ Not reported** | | | |
