# Supplementary material for: Assessing the level of evidence on transfer and transition in young people with chronic conditions: protocol of a scoping review
Source: Syst Rev. 2016 Sep 29;5:166. doi: 10.1186/s13643-016-0344-z (PMC5043611; doi:10.1186/s13643-016-0344-z)
Supplement: Additional file 6: — Flowchart of study designs. [file 13643_2016_344_MOESM6_ESM.docx]

**Additional file 6: Flowchart of study designs**

**ns**

**NO RANDOMNIZATION**

**RANDOMNIZATION**

## Flowchart study designs
